# Myocardial infarction with a preserved ejection fraction—the impaired function of the cardio-renal baroreflex

**DOI:** 10.3389/fphys.2023.1144620

**Published:** 2023-04-04

**Authors:** Lisa Pickny, Martin Hindermann, Tilmann Ditting, Karl F. Hilgers, Peter Linz, Christian Ott, Roland E. Schmieder, Mario Schiffer, Kerstin Amann, Roland Veelken, Kristina Rodionova

**Affiliations:** ^1^ Department of Internal Medicine 4—Nephrology and Hypertension, Friedrich-Alexander University Erlangen, Erlangen, Germany; ^2^ Department of Internal Medicine 4—Nephrology and Hypertension, Paracelsus Private Medical School Nuremberg, Nuremberg, Germany; ^3^ Department of Radiology, Friedrich-Alexander University Erlangen, Erlangen, Germany; ^4^ Department of Nephropathology, Friedrich-Alexander University Erlangen, Erlangen, Germany

**Keywords:** cardiac afferent neurons after myocardial infarction cardiac afferent innervation, renal innervation, neuronal cell culture, myocardial infarction, congestive heart failure, preserved ejection fraction

## Abstract

**Introduction:** In experimental myocardial infarction with reduced ejection fraction causing overt congestive heart failure, the control of renal sympathetic nerve activity (RSNA) by the cardio-renal baroreflex was impaired. The afferent vagal nerve activity under these experimental conditions had a lower frequency at saturation than that in controls. Hence, by investigating respective first neurons in the nodose ganglion (NG), we wanted to test the hypothesis that after myocardial infarction with still-preserved ejection fraction, the cardiac afferent nerve pathway is also already impaired.

**Material and methods:** A myocardial infarction was induced by coronary artery ligature. After 21 days, nodose ganglion neurons with cardiac afferents from rats with myocardial infarction were cultured. A current clamp was used to characterize neurons as “tonic,” i.e., sustained action potential (AP) firing, or “phasic,” i.e., <5 APs upon current injection. Cardiac ejection fraction was measured using echocardiography; RSNA was recorded to evaluate the sensitivity of the cardiopulmonary baroreflex. Renal and cardiac histology was studied for inflammation and fibrosis markers.

**Results:** A total of 192 neurons were investigated. In rats, after myocardial infarction, the number of neurons with a tonic response pattern increased compared to that in the controls (infarction vs. control: 78.6% vs. 48.5%; z-test, *p < 0.05), with augmented production of APs (23.7 ± 2.86 vs. 15.5 ± 1.86 APs/600 ms; mean ± SEM, *t*-test, *p < 0.05). The baseline activity of RSNA was subtly increased, and its control by the cardiopulmonary baroreflex was impaired following myocardial infarction: the fibrosis marker collagen I augmented in the renal interstitium.

**Discussion:** After myocardial infarction with still-preserved ejection fraction, a complex impairment of the afferent limb of the cardio-renal baroreflex caused dysregulation of renal sympathetic nerve activity with signs of renal fibrosis.

## Introduction

The cardio-renal baroreflex, whose afferent portion is contained within vagus nerve pathways, influences sodium and volume homeostasis by controlling renal sympathetic nerve activity (RSNA) (Rodionova et al., 2021b). In congestive heart failure after myocardial infarction, the cardiopulmonary baroreflex control of renal sympathetic nerve activity has been repeatedly reported to be markedly attenuated due to abnormalities in the afferent branch of the cardiopulmonary baroreceptor reflex (DiBona and Sawin, 1994b; Rostagno et al., 1999; Dunlap et al., 2019).

With respect to renal afferent innervation, it is known that it consists of a characteristic composition of tonic (high-activity) and phasic (low-activity) neurons, which shifts toward phasic neurons in nephritis and hypertension (Ditting et al., 2009; Rodionova et al., 2020b). With respect to the mentioned cardiopulmonary baroreflex, whose first neuron is located within the nodose ganglion (NG) (Linz and Veelken, 2002), it is not known if it comprises a characteristic pattern of tonic and phasic neurons. The directly measured afferent renal nerve activity decreased in nephritis, while the number of tonic neurons dropped significantly (Rodionova et al., 2020b). This could suggest that tonic neurons will be likewise reduced in the afferent portion of the cardiac baroreflex after myocardial infarction with ensuing congestive heart failure. Generally, one could speculate that fewer tonic neurons on afferent pathways might be a measure of a decreased afferent nerve activity *in vivo*. On the other hand, tonic and thus likely fast responding units might also subserve the sensitivity of afferent neural pathways. In this respect, it is interesting that the sensitivity or gain of the cardiac baroreflex is impaired after myocardial infarction (DiBona and Sawin, 1994b).

In a large group of patients with congestive heart failure, important subgroups such as heart failure with reduced ejection fraction (HFrEF) and heart failure with preserved ejection fraction (HFpEF) are distinguished (Paulus and Tschope, 2013; Mishra and Kass, 2021). The latter has been increasing during recent years, specifically among aging populations worldwide (Taqueti et al., 2018).

HFpEF accounts for approximately half of all patients with heart failure and more than half of heart failure-related hospitalizations (Owan et al., 2006; van Riet et al., 2016; Schulz and Schuster, 2022). Mortality associated with HFpEF is eight-fold higher than in unimpaired cardiac functions, and six-month mortality after the onset of initial symptoms is comparable to that of HFrEF (Owan et al., 2006; van Riet et al., 2016; Schulz and Schuster, 2022). The prevalence of HFpEF is increasing, mainly due to an aging population and an increasing burden of comorbidities, such as hypertension, diabetes, and obesity (Tsao et al., 2018).

The causes are diverse, the diagnosis is laborious, and the treatment options are less clear than those of other forms of congestive heart failure (Paulus and Tschope, 2013).

In light of these clinical and epidemiological data, it is urgently necessary that more experimental data in animals with more subtle alterations of myocardial ischemia become available to understand how early cardiac pathologies will influence the cardiovascular system and its neural regulators.

In light of these facts and questions, we wanted to test the hypothesis that, in rats after myocardial infarction with preserved ejection fraction, the portion of tonic neurons in the afferent branch of the cardiac baroreflex will decrease at the expense of phasic neurons. For the first time, we used a model of congestive HFpEF in the experimental animals (Cunningham et al., 2020). Such a model for a complex, not-well-understood disease such as HFpEF offers the advantage of an isolated focus on the heart with a higher degree of still functional heart tissues (Rodionova et al., 2021b). We investigated neurons with cardiac dendrites and projections from other sites. The function of the cardiac baroreceptor reflex was challenged with volume expansions to assess the control of sympathetic renal nerve activity (Rodionova et al., 2021b). Furthermore, renal collagen I expression was assessed to evaluate putatively subtle alterations of kidneys in rats after myocardial infarction due to a dysregulation of renal sympathetic nerve activity.

## Materials and methods

For the experiments, male Sprague Dawley rats (Ivanovas, Kisslegg, Germany) weighing 250–300 g were maintained in cages at 24°C ± 2°C. They were fed a standard rat diet (no. C 1000, Altromin, Lage, FRG) containing 0.2% sodium by weight and were allowed free access to tap water. All procedures performed on the animals were done in accordance with the guidelines of the American Physiological Society and in compliance with NIH Guide for Animal Care and Use in Laboratory Practice. They were approved by the local government agency (Regierung von Unterfranken).

### Induction of experimental myocardial infarction and congestive heart failure

A previously described technique (DiBona and Sawin, 1994b; Rodionova et al., 2021b; Omoto et al., 2022) involving ligation of a coronary artery was used to induce myocardial infarction. For all surgical procedures, rats were anesthetized with a mixture of 50% O_2_, 50% N_2_O, and 5% of isoflurane (Isofuran CP^®^ 1 mL/mL, CP Pharma, Burgdorf, Germany), while the latter was rapidly switched to a maintenance dose of ∼1.5%. Additional analgesia was ensured using buprenorphine (0.05 mg/kg, Temgesic; RB Pharmaceuticals, Berkshire, UK), given subcutaneously before and after surgery. An oral endotracheal tube was inserted, and mechanical ventilation with room air was instituted. The chest was opened at the fourth left intercostal space spreading the gap between the ribs with an inserted chest retractor. The pericardium was opened, and then the left anterior descending coronary artery was ligated. After the epicardial application of DiI for neuronal labeling (see later), the slightly twisted heart was returned to its normal position, and the thorax was closed with the removal of air. After recovering from anesthesia and removal from the ventilator, rats were returned to individual cages with free access to a normal sodium rat-pellet diet and tap water. The SHAM animals from the control were treated the same way without a ligation of the coronary artery.

### Echocardiographic assessment of the left ventricular function

Trans-thoracic echocardiography was performed using a 3100 Vevo^®^ Imaging Platform (Fujifilm Visual Sonics Inc., Toronto, Canada) with a 40 MHz linear array transducer (MS 400) (Biancardi et al., 2017; Stegmann et al., 2019) under light anesthesia (isoflurane 2%). Procedures were scheduled two days before the actual experiments that were begun three weeks after the cardiac surgery. Long-axis images at the level of the mitral and aortic valves were obtained from the longest length of the left ventricle. Short-axis views of the left ventricle at the level of chordae tendineae muscle were recorded perpendicular to its image to evaluate the cardiac parameters. Both anatomic imaging views were recorded in the brightness motion mode (m-mode), to assess the location and to obtain anatomical measurements. Two measurements of end-diastolic (EDV) and end-systolic volumes (ESV) were averaged. Automatic calculation using the parameters was obtained for the ejection fraction (EF) using the formula: EF = (ESV − EDV/EDV) × 100.

### Electrophysiology

#### Labeling of neurons with cardiac afferents from the nodose ganglion

To identify neurons with cardiac afferents in the nodose ganglion, in some of the experiments, we labeled these cells by applying the dicarbocyanine dye, 1,1′ dioleyl-3,3,3′ tetramethyl-indocarbocyaline methansulfonate (DiI) (D9 –DiI, DiI, 50 mg/mL in EtOH; Molecular Probes^®^, Darmstadt), epicardially to the junction of the great vessels in the heart (Linz and Veelken, 2002) and under anesthesia and analgesia as previously described. After the left anterior descending coronary artery was ligated and before closing the thorax, a fine glass cannula filled with DiI was used for respective epicardial application of the substance (5 µL of DiI 50 mg/mL).

#### Neuronal cell culture

Respective rats (controls or 3 weeks after coronary artery ligation) were anesthetized as previously described, the animals were decapitated, and both nodose ganglia dissected. Primary cultures of neurons from the nodose ganglia were obtained through mechanical and enzymatic dissociation by adapting protocols previously described (Linz and Veelken, 2002; Rodionova et al., 2020b). The ganglia were incubated with collagenase (4 mg/mL) in DMEM (PAA Laboratories GmbH, Linz, Austria) for 1 h in 5% CO_2_ at 37°C. Enzymatic dissociation was terminated by replacing collagenase containing DMEM with fresh DMEM+, a cultural medium containing 10% fetal calf serum (FCS), 1% penicillin/streptomycin, and 0.1% insulin. Tissue digestion was stopped using FCS. Ganglia were triturated using sterile Pasteur pipettes (Sigmacote^®^; Sigma-Aldrich, Munich, Germany) to dissociate individual cells. After centrifugation at 100 rcf, cells were resuspended in 10 mL DMEM+ and centrifuged once more. The pellet was resuspended in 1.8 mL DMEM+ and cells were plated on glass coverslips coated with poly-L-lysine. The neurons on coverslips were cultured normally in DMEM+ for one day before electrophysiological experiments.

To demonstrate that the labeled cells were neurons, all cells used for experimental procedures were tested for fast sodium currents during repolarization; these are a characteristic of the neuronal cells. Stained coverslips were viewed under epifluorescence to permit visualization of respective neurons before the experiment. Furthermore, a small laser beam (wavelength of 532 nm) powered by a storage battery was mounted on the patch clamp recording set-up. This equipment allowed for the detection of DiI-stained nodose ganglion cells during the experiments using respective optical filters.

#### Investigation of cultivated neurons

Patch clamp recordings were obtained from respective neurons (from nodose ganglia of rats with infarction and controls). Recordings were obtained within 30 h of plating.

In total, 192 neurons from six rats with myocardial infarction and six SHAM control rats were investigated.

Patch clamp recordings were obtained using a pipette solution containing 140 mM KCl, 5 mM NaCl, 2 mM MgCl_2_, 1 mM CaCl_2_, 2 mM Mg-ATP, 0.3 mM Na-GTP, 10 mM EGTA, and 10 mM HEPES (pH 7.4). Recordings were conducted in the whole-cell-mode. Patch pipettes were pulled from borosilicate-glass capillaries (GB150F-8P; Science Products, Hofheim, Germany) in a two-stage process using a microelectrode puller and a microforge (PP-830, Narishige, Tokyo, Japan) to adjust the opening diameter at a resistance of 2–4 MΩ.

Patch clamp recordings were obtained using an Axopatch 200B amplifier (Axon Instruments, Foster City, CA). Data were sampled at 5 kHz for voltage and 20 kHz for a current clamp and analyzed with pClamp^®^ 10.2 (Axon Instruments, Foster City, CA).

Only neurons with a resting membrane potential below −40 mV were measured. Cells that stained brightly with DiI under laser excitation (wavelength, 532 nM) were considered as cardiac afferent neurons. Non-cardiac neurons from the same cultures that showed no DiI staining at all were also tested (Linz et al., 2006). All recordings were done at room temperature, i.e., 22°C ± 2°C.

Cell capacitance was compensated manually, and cell parameters (size, capacitance, and resistances) were documented. To confirm vitality of each neuron, typical neuronal currents were examined using a voltage step protocol (step duration of 50 ms, from −100 mV to +60 mV in 17 steps with a 1-s delay). Neurons were identified by the presence of fast sodium currents during repolarization.

#### Current-clamp protocols—effects on cardiac neurons from rats with myocardial infarction and controls

To determine firing patterns of cardiac neurons from the nodose ganglia, we used a whole-cell current-clamp approach previously described (Sculptoreanu and de Groat, 2007; Ditting et al., 2009; Rodionova et al., 2020b). pClamp^®^ 10.2 (Axon Instruments, Foster City, CA) was used to control current-pulse generation, to record membrane potentials, and for off-line data analysis.

To start patch clamp recordings, coverslips containing the cardiac neuron’s ganglion were transferred to a laminar flow chamber placed on an inverted phase-contrast microscope (Wilowert, Hund, Germany). Action potential generation was induced using rectangular current-pulse injections as follows: a 5 ms pre-pulse, followed by a 600-ms lasting pulse with an inter-pulse delay of 100 ms, was delivered in three consecutive trains of increased intensity (40–400 pA, 400–4,000 pA, and 4,000–12,000 pA) in 10 consecutive steps (5.16 s, each). We categorized cardiac neurons from the nodose ganglion as “tonic” or “phasic” as described previously (Sculptoreanu and de Groat, 2007): Neurons generating five or more APs were defined as high-activity “tonic.” In contrast, neurons generating one to four APs were defined as low-activity “phasic.”

### Neurophysiology

#### Recordings of renal nerve activity

Three weeks after coronary artery ligature, in anesthetized rats with myocardial infarction and controls, polyethylene catheters were inserted in the arterial or venous femoral vessels and recording electrodes were implanted (Veelken et al., 1996; Rodionova et al., 2021b). Nine rats with myocardial infarction and six SHAM controls were eventually used for final evaluation.

#### First approach: Integration of RSNA

Recordings of left-sided RSNA were obtained as previously described (Kopp et al., 1987; Kopp et al., 1998; Ditting et al., 2007). Through a left lateral approach, renal nerve fibers were dissected and freed from connective tissue and placed on a bipolar electrode. The nerve signals were full-wave rectified and integrated over 1-s intervals with commercially available data acquisition and analysis software (SciWorks 7.2, DataWave Technologies, Loveland, CO, United States). After control periods of 15 min each, all rats received a volume challenge with saline for 15 min (10% of body weight). Thereafter, three further periods of 30 min each began for recovery while RSNA was continuously recorded. Eventually, 3 mg/rat of the ganglionic-blocking agent, trimetapham-camsylate (Hoffmann-La Roche, Basel, Switzerland), was administered to wipe out postsynaptic renal sympathetic nerve activity. The background activity was subtracted from the recorded activity throughout the experiment.

#### Second approach: baseline analysis of renal sympathetic nerve activity

**FIGURE 1:**
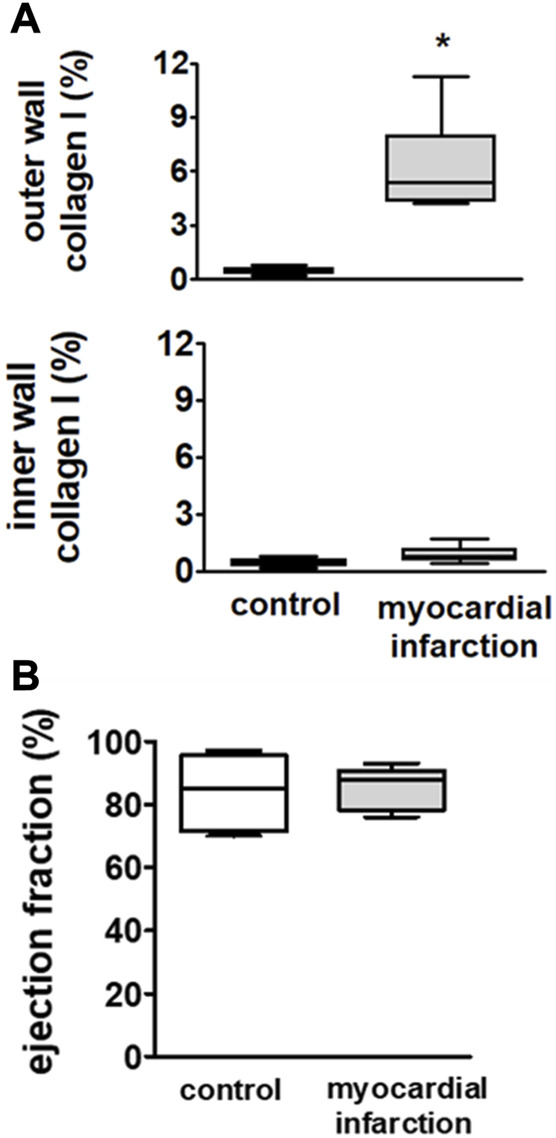
Upper panel **(A)**: infiltration of macrophages in the outer wall and the inner wall of the left ventricle in SM controls (*n* = 6) and rats with myocardial infarction (*n* = 9). Rats after myocardial infarction exhibited a considerable increase of macrophages in the outer wall of the left ventricle, suggesting a non-transmural infarction. Lower panel **(B)**: echocardiographically assessed ejection fractions (EFs) were not different among rats after myocardial infarction (*n* = 9) and controls (*n* = 6). Hence, no overt congestive heart failure could be observed in rats after myocardial infarction. Kruskal–Wallis one-way ANOVA on ranks was used with Dunn’s *post*-*hoc* method. Rats were taken from the groups of rats with myocardial infarction or SHAM controls.

In addition to integration of RSNA over 1-s intervals, RSNA-burst analysis was carried out using a software-based programmable algorithm (SciWorks 7.2). Continuous nerve activities lasting longer than three single spikes (i.e., >8 ms) were detected as bursts, followed by silent periods with some single spikes (Rodionova et al., 2020b). Approximately 10,000 bursts/rat were analyzed, and the burst amplitude, burst duration, burst area (i.e., duration integral), and the burst frequency were measured (Figure 1A). Due to ganglionic blockade (10 mg/kg trimetaphan camsylate iv), efferent bursting activity disappeared but afferent spikes were unmasked and could be counted using a programmed algorithm (using SciWorks 7.2) over a 1-min interval to calculate the afferent renal nerve activity (ARNA) in terms of the spike frequency (Figure 1B) (Rodionova et al., 2020b).

#### Arterial and venous lines: bladder cannula

Polyethylene catheters (PE-10) were inserted into the right femoral artery and vein after exposure *via* a small incision in the right groin region. Catheters were inserted into the vessels with the help of a micro forceps (Dumont Nr. 5) under a dissection microscope (Wild Leitz, Germany). The arterial femoral catheter was connected to a pressure transducer (Gould Statham P23Db, connected to a pressure processor type 13-4615-52, Gould Instrument Systems, Valley View, OH), to record the mean arterial blood pressure (MAP) and the heart rate (HR). Femoral venous lines were used for the administration of substances and volume expansion with saline (Rodionova et al., 2020b). A PP 50 tube was inserted into the bladder *via* a 2 mm suprapubic skin incision.

We used a respective infusion pump for volume expansion and background administration of saline. The weight of each rat was determined acutely on the day of the experiments. Exactly 5% of the body weight in grams was converted into milliliters of saline solution, and the corresponding amount of physiological saline solution was then drawn into an infusion syringe. The infusion rate varied slightly depending on the infusion volume, which was always divided by 15 to set an appropriate infusion rate in milliliters per minute at the infusion pump. The basal background infusion of saline was 6 μL/min before and after the actual volume expansion*.*

### Kidney and heart histology

For evaluation of histology, renal and cardiac tissues were fixed overnight in paraformaldehyde solution, dehydrated in increasing concentrations of methanol, followed by 100% isopropanol, and then were embedded in paraffin. Four-micrometer sections were stained with periodic acid-Schiff (Veelken et al., 2008).

#### Immunohistochemistry of the kidney

After deparaffinization, peroxidase activity was blocked in sections of the paraformaldehyde-fixed paraffin-embedded tissue with 3% H_2_O_2_ for 20 min. A mouse monoclonal antibody (mAb) detecting the macrophage marker ED-1 (Serotec, Biozol, Eching, Germany), and a goat polyclonal antibody (PAb) to collagen I (Southern Biotechnology Associates, Birmingham, AL, United States) were used in respective dilutions of 1:50 for collagen I and 1:250 for ED-1. Immunostaining was carried out using a 0.1% diaminobenzidine tetrahydrochloride/0.02% H_2_O_2_ detection system (Vector Laboratories, Burlingame, CA).

Interstitial proliferating cell nuclear antigen (PCNA) (Santa Cruz Biotechnologies, Heidelberg, Germany) or ED-1-positive cells were counted in 20 high-power (magnification ×400) cortical views per section and expressed as cells per mm^2^. Glomerular collagen type I staining was measured morphometrically using image analysis software (Metaview, Visitron Systems, Puchheim, Germany), and the stained area was expressed as a percentage of the total area under investigation.

#### Immunohistochemistry of the heart

After deparaffinization, peroxidase activity was blocked again in sections of the paraformaldehyde-fixed paraffin-embedded tissue with 3% H_2_O_2_ for 20 min. As described for the kidney, a goat polyclonal antibody to collagen I (Southern Biotechnology Associates, Birmingham, AL, United States) was used in respective dilutions, and immunostaining was carried out as previously described. Downstream of the ligated left anterior descending coronary artery, collagen type I staining was measured generally again as described for the kidney and expressed as a percentage of the total area of investigation. Specifically, we analyzed collagen I expression in the outer cardiac wall adjacent to the surface of the right and left ventricles separately from the inner cardiac wall adjacent to the chamber of the right and left ventricles in rats with myocardial infarction and SHAM controls.

### Data analyses

All data were tested for normality using a Kolmogorov–Smirnov test. T-tests were used for comparison of two groups or treatments, if data were normally distributed; otherwise, non-parametric testing (Mann–Whitney rank sum test) was performed. More than two groups or treatments at different points in time were evaluated by one-way ANOVA with a Student–Newman–Keuls *post*-*hoc* test, if data were normally distributed; otherwise, Kruskal–Wallis one-way ANOVA on ranks was used with Dunn’s *post*-*hoc* method. Statistical significance was defined as *p* < 0.05 (two sided). The z-test was used to test for significant differences in the frequency distribution of firing characteristics of neurons from the nodose ganglion (e.g., tonic vs. phasic; myocardial infarction vs. control). Data are presented as group means ± SD or range, or as box–whisker plots, in which the box boundaries denoted the first and third quartiles, and whiskers indicated the fifth and 95th percentiles. SigmaPlot 8.0 and SigmaStat 3.5 (Systat Software, Erkrath, Germany) were used for statistical analysis and graphical display.

## Results

The mean body weight of animals with heart failure (2,809 g) was not different from that of the controls (30,610 g) (*n* = 17).

### Blood pressure and heart rate

Rats with myocardial infarction and controls did not show differences in mean arterial blood pressure [rats with myocardial infarction: 98 mmHg (87–115) vs. controls: 102 mmHg (70–115); (range: minimum–maximum)] or the heart rate [rats with myocardial infarction: 406 bpm (389–428) vs. controls: 412 bpm (391–443); (range: minimum–maximum)]. However, due to volume expansion, MAP decreased significantly in both groups to the same extent and did not recover to baseline values throughout the experimental protocol (Figure 7A). The heart rate was not specifically influenced by volume loading (Figure 7B).

### Heart after myocardial infarction

Downstream of the ligated left anterior descending coronary artery, we observed significant increases in collagen I in the outer cardiac wall adjacent to the surface of the right and left ventricles. This increase could be neither observed in the inner cardiac wall adjacent to the chamber of the right and left ventricles nor anywhere in respective areas of the heart from the SHAM controls (Figure 1A). These findings suggest the development of a non-transmural infarction after ligature of the respective coronary artery.

There were no differences in ejection fraction among rats after myocardial infarction and controls (Figure 1B). Hence, the rats did not suffer from overt congestive heart failure.

### Dil-labeled cells *in situ* and in culture

In the nodose ganglion, Dil-labeled neurons could be already identified *in situ* although a clear pattern of distribution of neurons with cardiac afferents could not be seen. A small fraction of these cells were brightly labeled with Dil. The cells clearly labeled were classified as putative nodose ganglion cells with cardiac afferents; the cells without any response to laser light were regarded as neurons with non-cardiac afferents.

**FIGURE 2:**
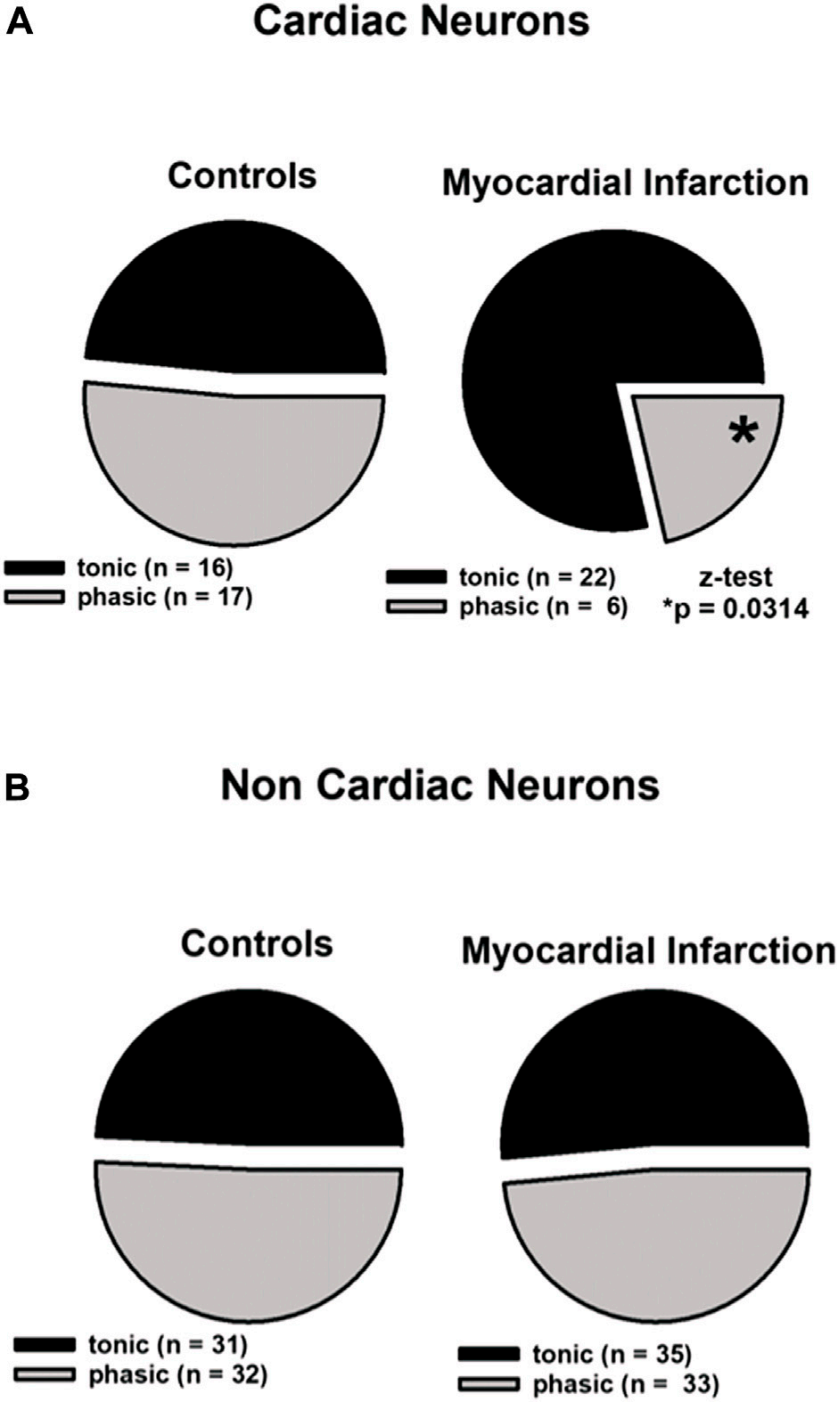
Myocardial infarction altered the firing pattern of cardiac afferent neurons. In the cardiac and non-cardiac samples, the tonic and phasic fractions did not differ in size regarding controls. Upper panels **(A)**: in cardiac neurons (*n* = 61), a significant increase of the tonic fraction was observed in animals after myocardial infarction 78.6% [*(22 out of 28; myocardial infarction) vs. 48.5% (16 out of 33; control), *p* < 0.05]. Lower panels **(B)**: in non-cardiac neurons (*n* = 131), myocardial infarction did not lead to significant changes of the tonic fraction [(35 out of 68; myocardial infarction) vs. (31 out of 63; control)]. Statistical analyses were performed using the z-test. Neurons were investigated in the current clamp mode of patch clamp equipment.

After myocardial infarction, the occurrence of tonic firing in cardiac neurons upon electrical stimulation was significantly increased (Figure 2).

Firing patterns of 61 cardiac neurons and 131 non-cardiac neurons from the NG were analyzed and classified as high-activity “tonic” or low-activity “phasic” neurons as aforementioned. Cardiac NG neurons after myocardial infarction showed a significant more frequent tonic firing pattern (78.6% vs. 48.5%, z-test, *p < 0.05) and lower occurrence of phasic firing as compared to healthy controls (21.4% vs. 51.5%, z-test, *p < 0.05) (Figure 2, upper panels). Non-cardiac NG neurons were not affected by myocardial infarction in terms of firing pattern and showed a similar percentage of tonic firing as in controls (Figure 2, lower panels).

**FIGURE 3:**
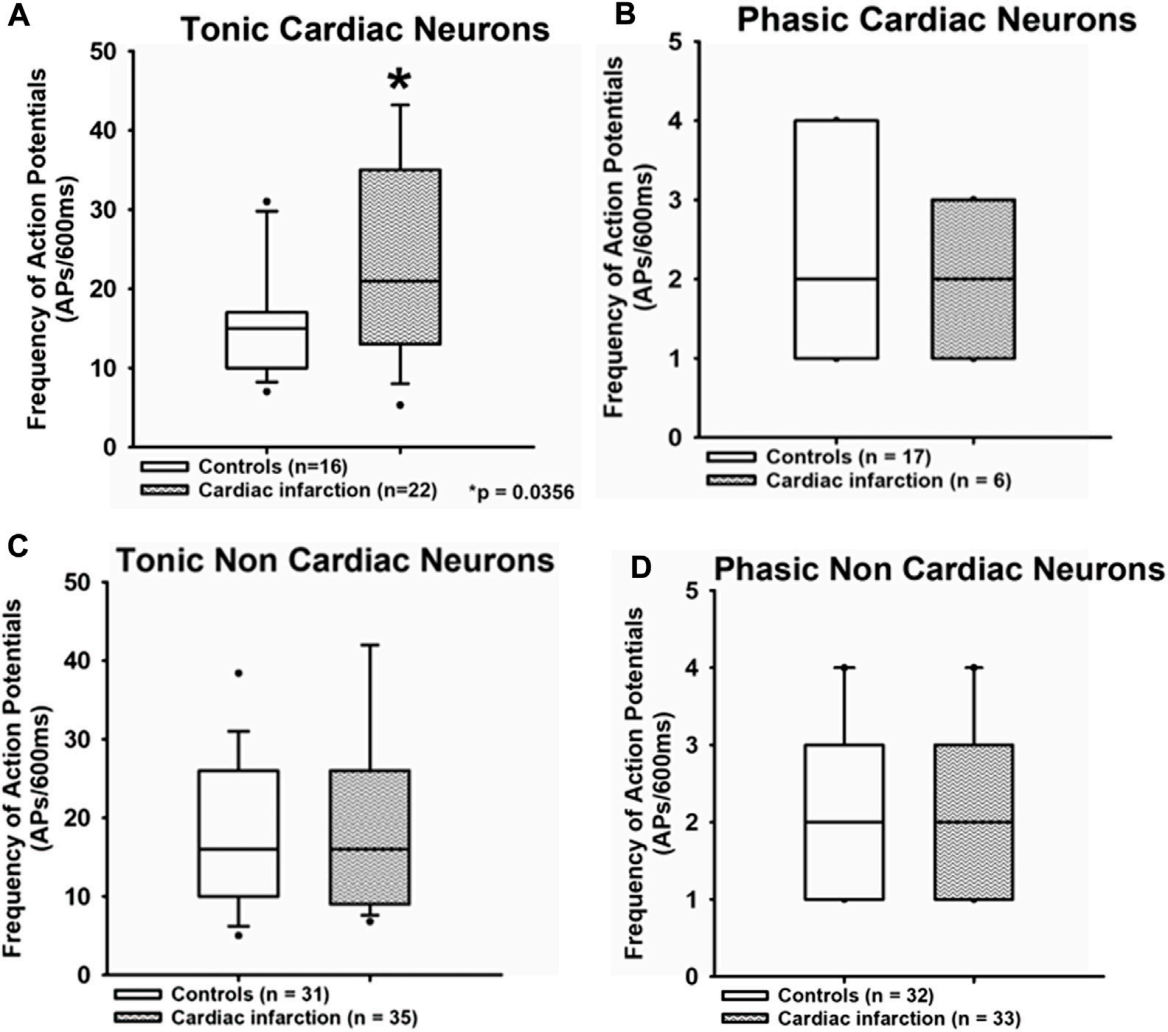
Myocardial infarction altered the frequency of action potential firing in tonic neurons. The frequency of action potentials (APs/600 ms) due to current injection was presented. Upper panels **(A,B)**: the frequency of action potentials was significantly increased in the tonic cardiac neurons of rats with myocardial infarction [cardiac control: 15 APs/600 ms (10.8; 16.3) vs. cardiac after myocardial infarction: 21 APs/600 ms (14; 38.5)] *p < 0.05. Lower panels **(C,D)**: neurons with a phasic firing pattern exhibited no significant differences in frequencies of action potentials in rats with myocardial infarction. Shown are the medians, the 25th/75th (box boundaries) and the 10th/90th percentiles (black dots). Kruskal–Wallis one-way ANOVA on ranks was used with Dunn’s *post*-*hoc* method. Neurons were investigated in the voltage clamp mode of patch clamp equipment.

After myocardial infarction, the frequency of action potentials upon electrical stimulation in tonic cardiac NG neurons was significantly increased (Figure 3).

The frequency of action potentials upon stimulation in cardiac tonic NG neurons was significantly increased after myocardial infarction. Data are displayed as median (25th and 75th percentiles) [cardiac control: 15 APs/600 ms (10.8; 16.3) vs. cardiac after myocardial infarction: 21 APs/600 ms (14; 38.5), *p < 0.05]. No significant differences were observed in a non-cardiac tonic group [non-cardiac control: 16 APs/600 ms (10; 25.1) vs. non-cardiac after myocardial infarction: 16 APs/600 ms (9.25; 26)]. There are also no differences between all groups of phasic neurons [cardiac control: two APs/600 ms (1; 4) vs. cardiac after myocardial infarction: two APs/600 ms (1; 3); non-cardiac control: two APs/600 ms (1; 3] vs. non-cardiac after myocardial infarction: two APs/600 ms (1; 3)].

**FIGURE 4:**
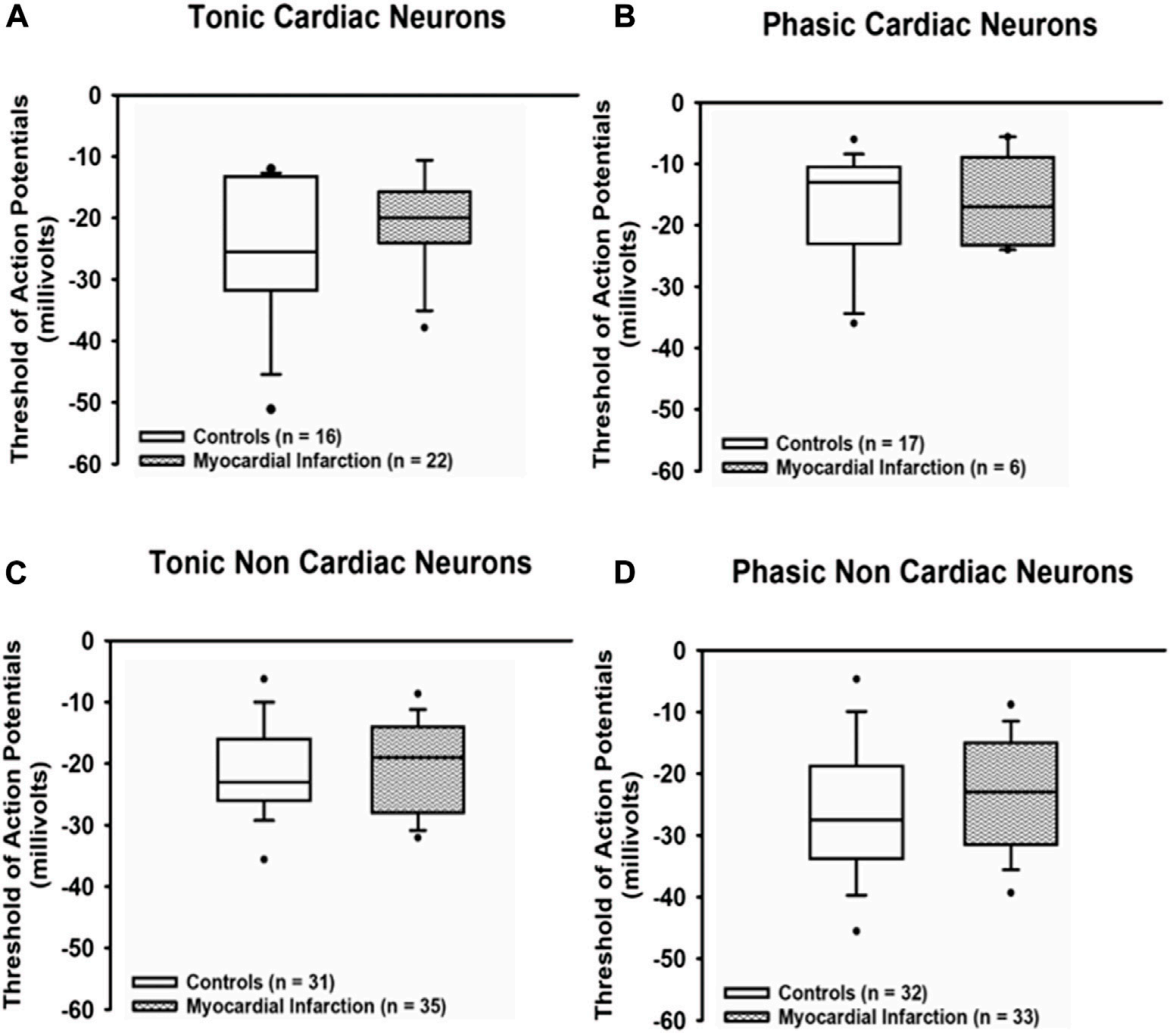
Myocardial infarction did not affect threshold potentials. Threshold potentials (mV) of action potentials due to current injection in all groups of neurons [upper panels **(A,B)**: cardiac; lower panels **(C,D)**: non-cardiac] were not significantly different among the various groups of neurons. Shown are the medians, the 25th/75th (box boundaries) and the 10th/90th percentiles (black dots). Kruskal–Wallis one-way ANOVA on ranks was used with Dunn’s *post*-*hoc* method. Neurons were investigated in the voltage clamp mode of patch clamp equipment.

The action potential threshold (Figure 4) in all samples of neurons investigated proved to be not different from control neurons after myocardial infarction [tonic response pattern—cardiac control: −25.5 mV (−31.5; −13.5) vs. cardiac after myocardial infarction: −20 mV (−24; −16); non-cardiac control: −23 mV (−26; −16.1) vs. cardiac after myocardial infarction: −19 mV (−27.8; −14.3); phasic response pattern—cardiac control: −13 mV (−22.5; −10.75) vs. cardiac after myocardial infarction: −17 mV (−23; −10); non-cardiac control: −27.5 mV (−33.5; −19.5) vs. cardiac after myocardial infarction: −23 mV (−31.3; −15)].

**FIGURE 5:**
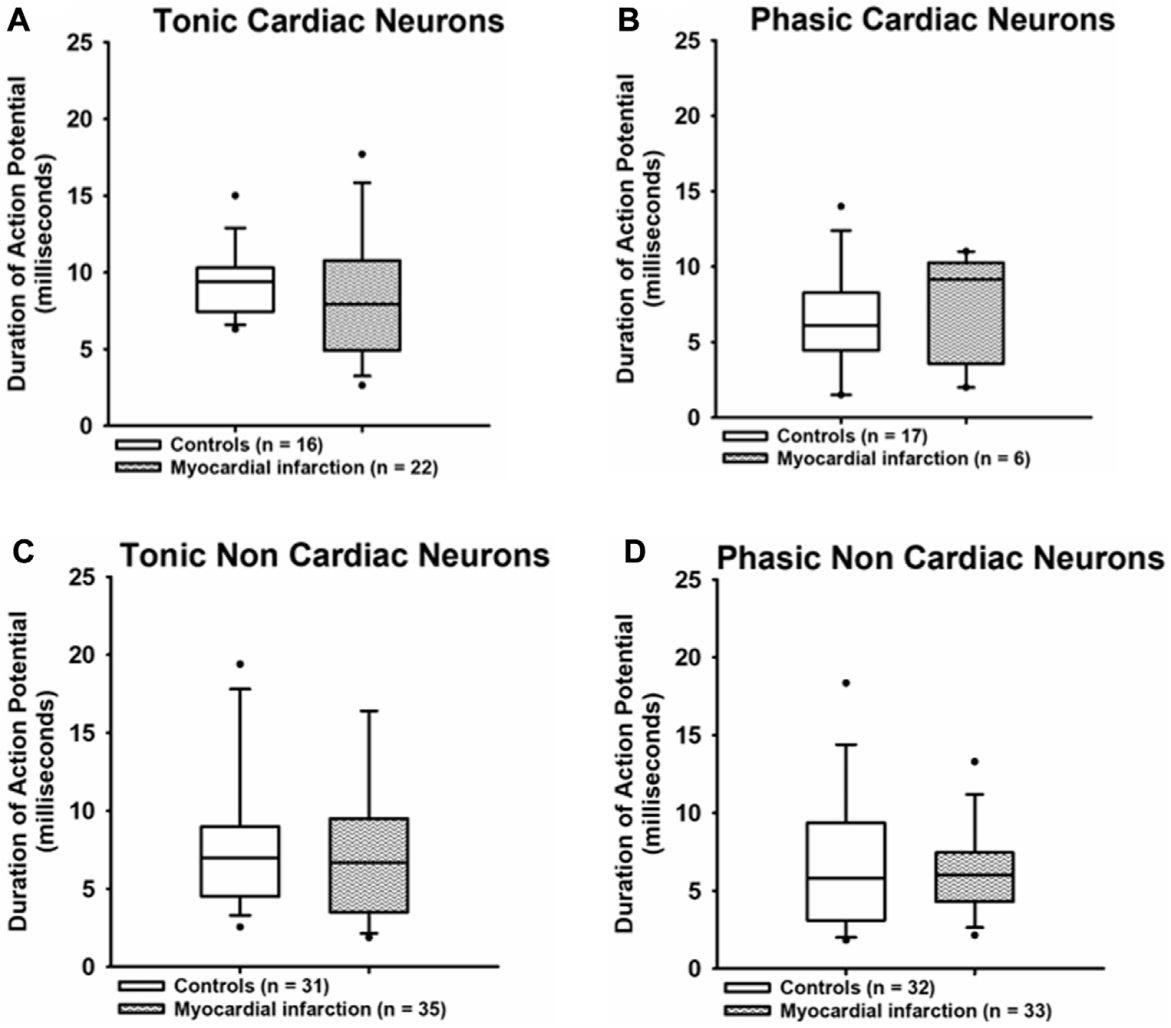
Myocardial infarction did not affect the duration of action potentials. The durations (ms) of action potentials measured at the threshold potential are presented. The duration of action potentials due to current injection in all groups of neurons [upper panels **(A,B)**: cardiac; lower panels **(C,D)**: non-cardiac] were again not significantly different among the various groups of neurons. Shown are the medians, the 25th/75th (box boundaries) and the 10th/90th percentiles (black dots). Kruskal–Wallis one-way ANOVA on ranks was used with Dunn’s *post*-*hoc* method. Neurons were investigated in the voltage clamp mode of patch clamp equipment.

The duration of action potentials (Figure 5) was likewise unaffected in any group of neurons investigated after myocardial infarction as compared to neurons from healthy control rats [tonic response pattern—cardiac control: 9.4 ms (7.45; 10.2) vs. cardiac after myocardial infarction: 7.95 ms (5.1; 10); non-cardiac control: 7 ms (4.63; 8.88) vs. non-cardiac control after myocardial infarction: 6.7 ms (3.8; 9.5); phasic response pattern—cardiac control: 6.1 ms (4.68; 8.23) vs. cardiac control after myocardial infarction: 9.15 ms (4.1; 10); non-cardiac control: 5.8 ms (3.2; 9.25) vs. non-renal after myocardial infarction: 6 ms (4.45; 7.43)].

**FIGURE 6:**
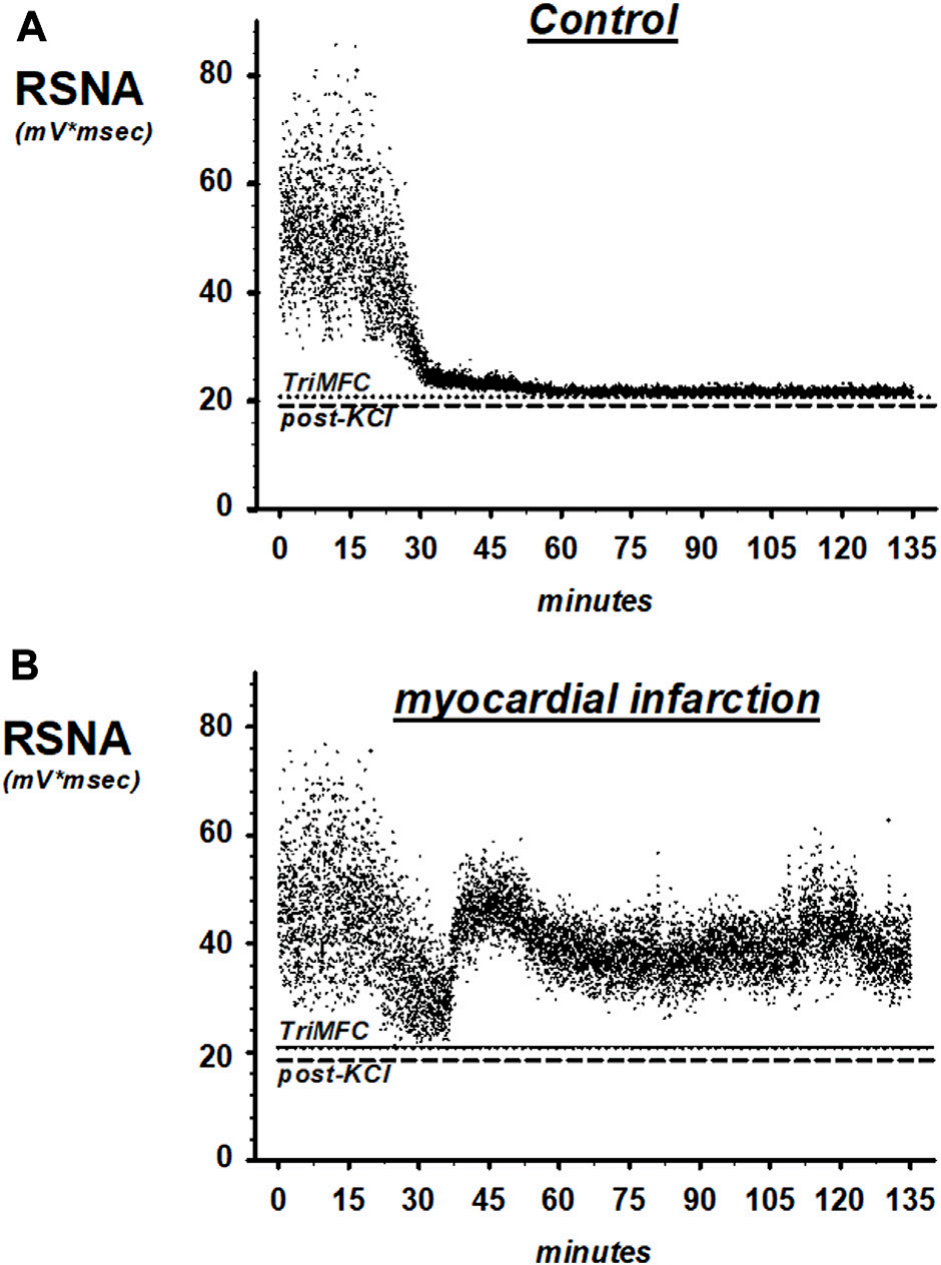
Representative recordings of renal sympathetic nerve activity (RSNA) in SHAM controls **(A)** and rats with myocardial infarction **(B)**. TrimMFC with dotted line represents the noise level after the administration of the ganglionic-blocking agent trimetapham-camsylate and post-KCL with dashed line after injecting potassium-chloride at the end of the experiments.

**FIGURE 7:**
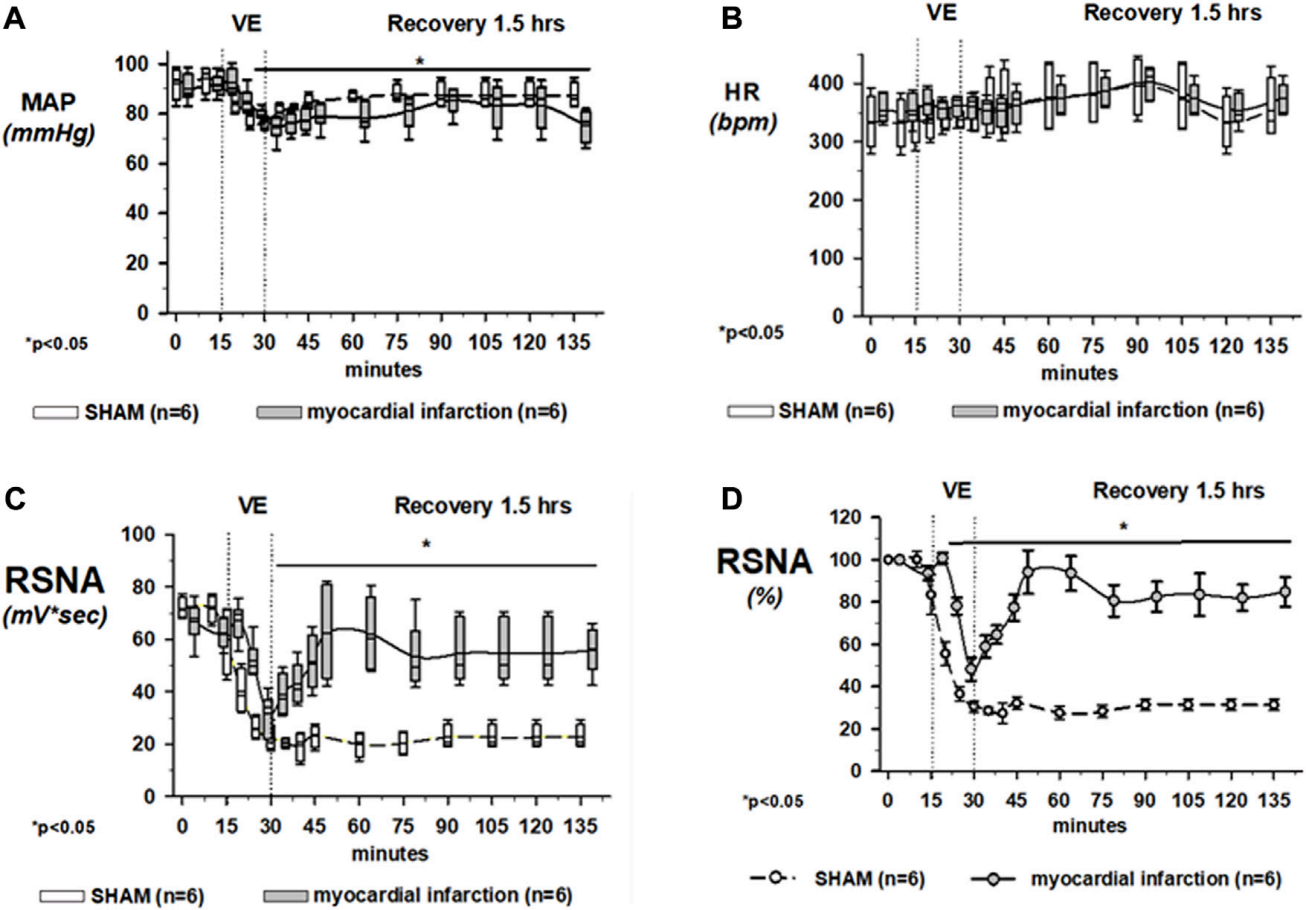
Effects of volume loading on the mean arterial blood pressure (MAP) and the heart rate (HR). Upper panel **(A)**: volume expansion lowered the mean arterial blood pressure in all groups slightly but significantly. Upper panel **(B)**: heart rate was not specifically influenced by volume loading. Volume loading unmasked renal sympathetic dysregulation in rats after myocardial infarction without overt congestive heart failure (cardiopulmonary baroreflex). Lower panel **(C)**: effects of 30-min volume expansion (VE) with saline (10% body weight) on renal sympathetic nerve activity (RSNA) in rats with coronary artery ligature (myocardial infarction; dotted line; *n* = 9) or control animals (control; solid line; *n* = 6). All data are presented as box and whiskers. *p < 0.05. Asterisks represent significant differences from RSNA baselines. Lower panel **(D)**: Raw data of the integrated renal sympathetic nerve recordings are normalized, and the changes are presented in present change from baseline. Using this way of presentation, in rats with myocardial infarction, renal sympathetic nerve activity was no longer suppressed to the same degree as in controls at the end of the 30-min volume expansion. Data are presented as mean ± SEM since data expressed in percent are normally distributed. *p < 0.05. Asterisks represent significant differences from RSNA baselines. In any case, rats with coronary artery ligature exhibited a distinctly different response pattern of RSNA to volume expansion as compared to that of normal controls. Kruskal–Wallis one-way ANOVA on ranks was used with Dunn’s *post*-*hoc* method. For panel **(D)**, statistical analysis was done using one-way ANOVA with a Student–Newman–Keuls *post hoc*. Rats were taken from the groups of rats with myocardial infarction or SHAM controls that were exposed to volume expansion.

Control of renal sympathetic nerve activity was impaired 3 weeks after myocardial infarction without overt congestive heart failure (Figure 6; Figures 7C, D).

Figure 6 shows original traces of renal sympathetic nerve recordings from rats with myocardial infarction and the SHAM controls. Each dot in the scatter plot represents a 1-s RSNA integral.

Mean changes of RSNA in rats with coronary artery ligature and control animals during volume expansion and recovery are shown in Figure 7C, showing raw integrated data in mV*sec and Figure 7D with normalized data, in which changes are now presented as percent change from the baseline. In rats with coronary artery ligature and controls, the maximal decrease in renal sympathetic nerve activity occurred at the end of the volume expansion of 15 min. There was no significant difference in the maximum depression of renal sympathetic nerve activity when we analyzed the raw integrated data. However, if we normalized our data, the maximum depression of RSNA was significantly less in rats with myocardial infarction at the end of the 15-min volume expansion period as compared to control animals.

In the first 30 min after cessation of the volume expansion, renal sympathetic nerve activity increased significantly above the activity of healthy controls in rats with coronary artery ligature and remained there for the duration of the recovery period. In control animals, however, renal sympathetic nerve activity remained depressed for the entire 1.5-h recovery period. In this latter respect, there were no differences regardless of analyzing raw or normalized data.

**FIGURE 8:**
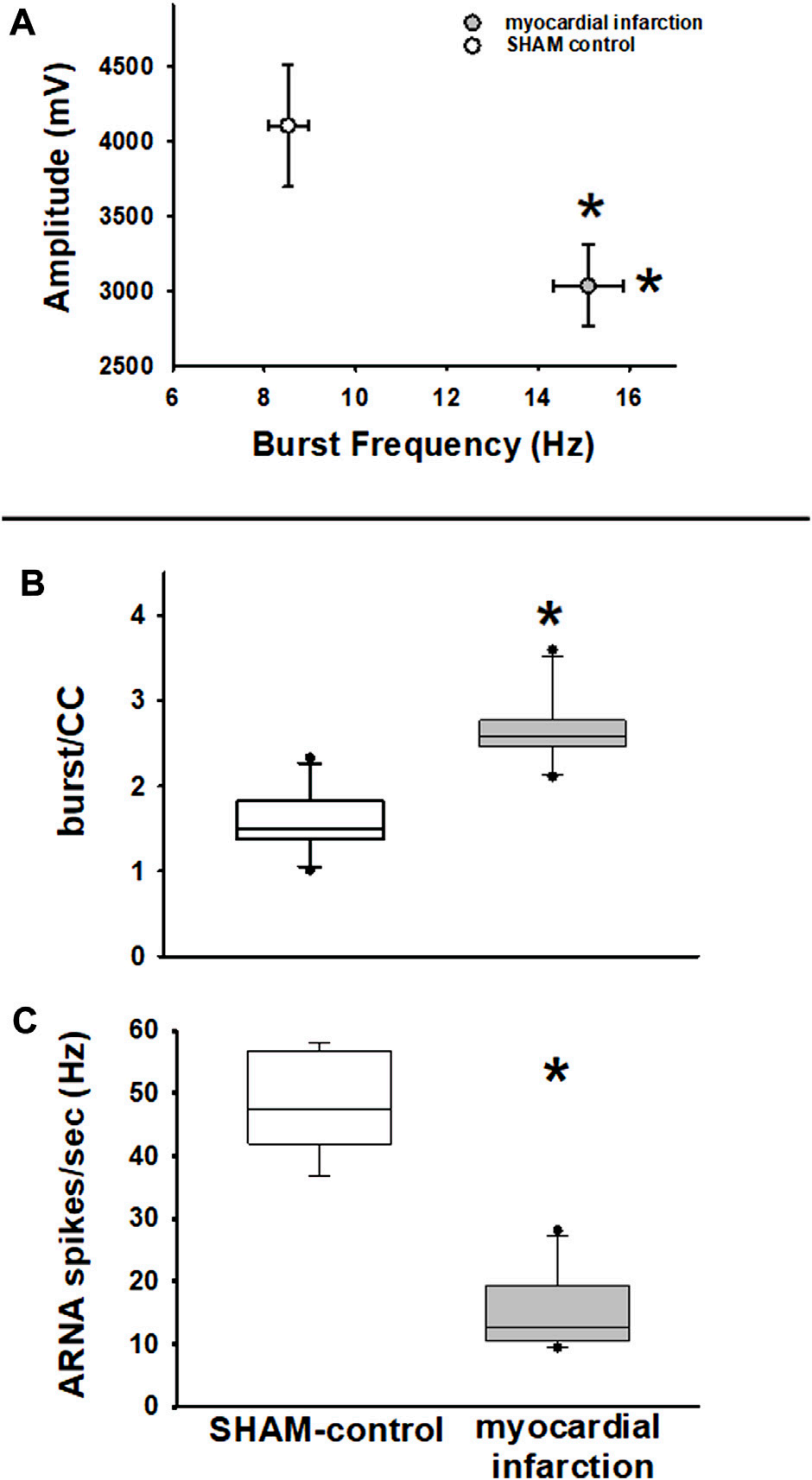
Efferent renal sympathetic nerve activity—frequency and amplitude. Upper panel **(A)**: renal sympathetic nerve activity (RSNA) burst amplitude versus RSNA burst frequency. RSNA burst frequency is higher in rats with myocardial infarction (*n* = 11) versus SHAM controls (*n* = 11, *p < 0.05), while RSNA burst amplitudes are lower (myocardial infarction vs. SHAM control, *p < 0.05, Kolmogorov–Smirnov test passed, *t*-test). Efferent renal sympathetic nerve activity and afferent renal nerve activity. Middle panel **(B)**: renal sympathetic nerve activity burst count per cardiac cycle (burst/CC) is higher in nephritic rats than in SHAM controls (myocardial infarction versus control, *p < 0.05, Kolmogorov–Smirnov test passed, *t*-test). Lower panel **(C)**: afferent renal nerve activity spike frequency (spikes/s) is lower in rats with myocardial infarction than in controls (myocardial infarction vs. control, *p < 0.001, Kolmogorov–Smirnov test passed, *t*-test).

Further analyses of baseline renal nerve activity are shown in Figure 8.:

Renal nerve activity was not only evaluated by integration of RSNA raw signal as previously described but also in terms of burst analysis of stored raw data as described in the Methods section. This analysis method is only viable for baseline data not for data that are dynamically changing due to an intervention.

With respect to RSNA, it turned out that the classical RSNA integration method did miss subtle differences of baseline RSNA between rats suffering from myocardial infarction and controls. A similar finding has been previously described in a nephritis model (Rodionova et al., 2020b). Specifically, the burst-analysis method showed that burst frequency in controls was lower, while burst amplitudes were higher than in rats after myocardial infarction (Figure 8A). Analysis of burst area data showed similar results. This finding was notably masked by integration of RSNA (Figures 7C, D). Furthermore, burst frequency in rats with myocardial infarction not only turned out to be higher in the time-based analysis but these rats also showed a higher burst count per cardiac cycle (Figure 8B). Hence, this analysis revealed a higher sympathetic nerve activity in rats after myocardial infarction.

Transient ganglionic blockade using trimetaphan camsylate iv completely abolished efferent RSNA bursting. Thus, ARNA could be analyzed. In rats with myocardial infarction and renal histologic changes, as outlined below, ARNA was considerably lower than that of controls (Figure 8C).

**FIGURE 9:**
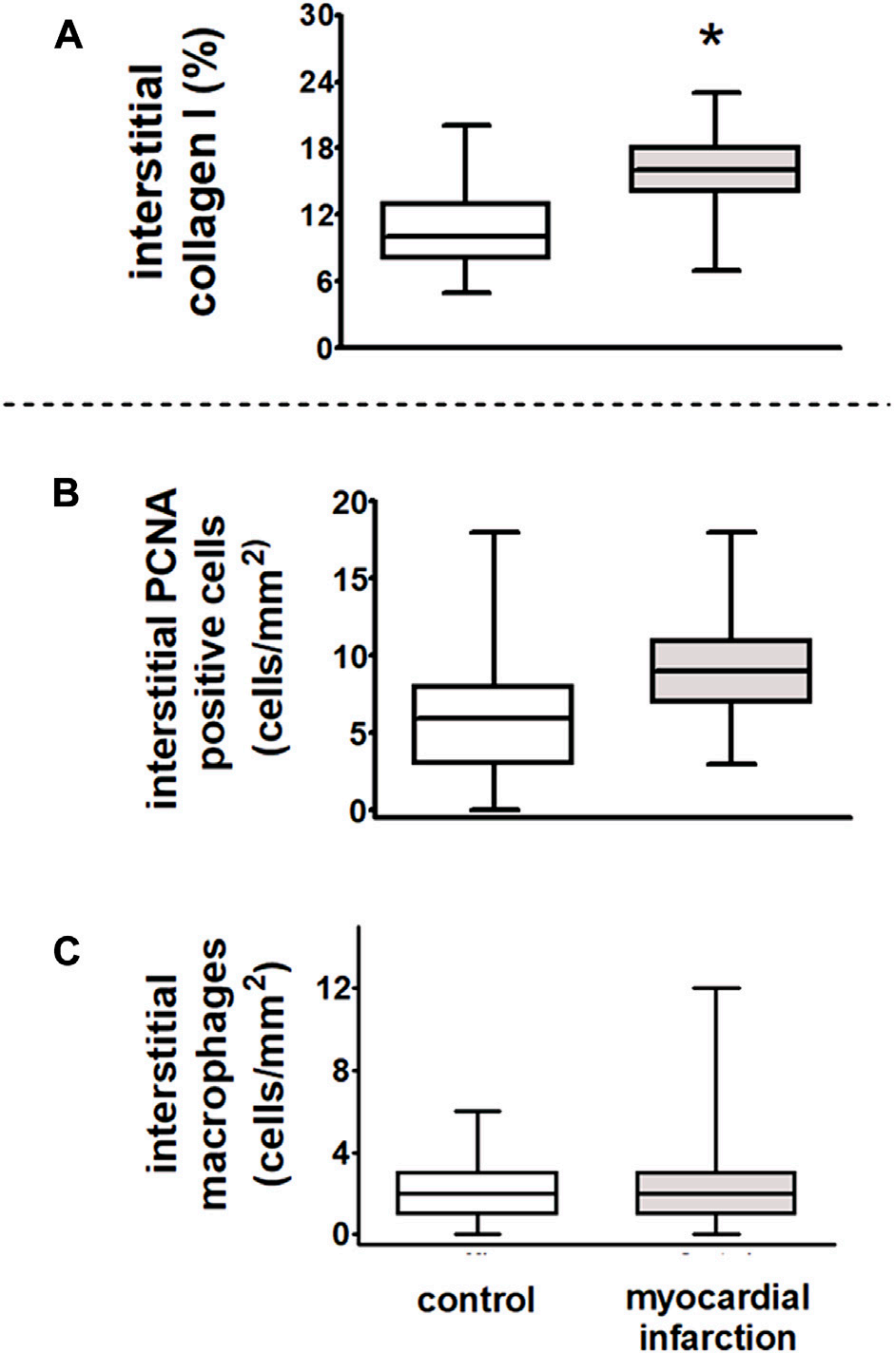
Dysregulation of renal sympathetic nerve activity and increase in collagen I expression after myocardial infarction in rats. After myocardial infarction, the fibrosis marker collagen I was increased in rats with myocardial infarction (*n* = 9) as compared to controls (*n* = 6). **(A)** Interstitial collagen type I (in % interstitial area, myocardial infarction versus controls, *p < 0.05). Myocardial infarction did not influence inflammatory infiltration of the renal interstitium as compared to control animals. **(B)** Interstitial proliferating cell nuclear antigen (PCNA)-positive cells (in cells/mm^2^, myocardial infarction versus controls, *n* = six per group); **(C)** interstitial macrophages (in cells/mm^2^, myocardial infarction versus controls, *n* = six per group). Kruskal–Wallis one-way ANOVA on ranks was used with Dunn’s *post*-*hoc* method. Rats were taken from the groups of rats with myocardial infarction or SHAM control.

In kidneys of rats after myocardial infarction, the interstitial collagen I expression was increased (Figure 9).

In rats after myocardial infarction with HFpEF that exhibited an already impaired cardiopulmonary baroreflex with dysregulation of the renal sympathetic nerve activity, we observed a significant increase in interstitial collagen I (Figure 9A). However, neither an increase of ED-1-positive macrophages nor PCNA staining as a marker of cell proliferation revealed interstitial differences among rats after myocardial infarction and controls. Hence, there were no signs of further inflammatory or sclerotic developments (Figures 9B, C).

## Discussion

In accordance with our assumptions, we could demonstrate for the first time that in samples of first neurons from the nodose ganglia, a separation in tonic and phasic neurons existed as frequently described for neurons of respective dorsal root ganglia in the afferent renal pathway (Rodionova et al., 2021a; Rodionova et al., 2020a).

In sharp contrast to our hypothesis, the portion of tonic versus phasic neurons increased (myocardial infarction vs. SHAM controls: 78.6% vs. 48.5%, z-test, *p <* 0.05) instead of decreased after myocardial infarction, although our *in vivo* experiments with direct sympathetic nerve recordings suggested an impaired control of sympathetic renal nerve activity after volume expansion, as previously described in projects with congestive heart failure after experimental myocardial infarction (DiBona and Sawin, 1994b; Rodionova et al., 2021b). The portion of phasic neurons decreased accordingly (myocardial infarction vs. SHAM controls: 21.4% vs. 51.5%, z-test, *p <* 0.05).

Furthermore, the frequencies of action potential induced in tonic cardiac neurons of rats with myocardial infarction by current injections were significantly higher than those of controls. Hence, although our *in vivo* experiments suggest a decreased control of RSNA by the afferent limb of the cardiac baroreflex, the first neurons of the afferent limb exhibited an increased sensitivity to stimuli. The likely explanation for this initially paradoxical finding might be that while *in situ*, in experimental animals with myocardial infarction, the neurons received less input from the affected hearts than in controls; they increased their sensitivity to compensate for this. Others also demonstrated an impairment of the afferent limb of the cardiac baroreflex and thus a reduced control of renal sympathetic nerve activity (DiBona and Sawin, 1994a). However, our findings suggest that those neurons which still have the ability to control sympathetic nerve activity become more sensitive to stimuli. This might explain the increasing portion of tonic-firing afferent neurons and also their higher firing frequencies after myocardial infarction.

The measured ejection fractions in rats were comparable to the data reported by others (Rahimi Kakavandi et al., 2022). However, we could detect a highly significant increase in collagen I in the outer wall of the ventricles that occurred downstream the ligated coronary artery (Figure 8, upper panel) proving a non-transmural infarction (Freifeld et al., 1983).

Volume expansion is a standard stimulus of the cardiopulmonary baroreflex. It has been known for quite some time that the control of renal sympathetic nerve activity by this reflex mechanism is impaired in congestive heart failure after myocardial infarction (DiBona and Sawin, 1994b; Dibner-Dunlap et al., 1996; Rostagno et al., 1999; Dunlap et al., 2019; Rodionova et al., 2021b). However, to the best of our knowledge, we are the first to report an impaired neurogenic cardio-renal axis already in circumstances with preserved cardiac output after experimental myocardial infarction. Hence, even if congestive heart failure with a reduced ejection fraction is absent, vagal cardiopulmonary baroreflex control of renal sympathetic nerve activity might be impaired much earlier in the development of left ventricular dysfunction than previously assumed (Dunlap et al., 2019). When we applied a strong stimulus, such as volume expansion, to the cardiopulmonary baroreceptors of animals with myocardial infarction, they were still, but barely, able to suppress sympathetic activity during the actual volume expansion, but when the strong stimulus of the infusion phase wore off, the signals transmitted to the central nervous system by the baroreceptors are no longer sufficient to properly downregulate renal sympathetic activity. With a more detailed analysis of single nerve bursts of the baseline, we could also detect an increased basal level of sympathetic activity in our model of heart failure with HFpEF.

*In vivo*, cardiac afferent nerve fibers have been investigated repeatedly in experimental congestive heart failure after myocardial infarction (DiBona and Sawin, 1994b; Dibner-Dunlap et al., 1996; Rostagno et al., 1999; Dunlap et al., 2019; Rodionova et al., 2021b). A very diligently elaborated paper on the topic has been published by DiBona and Sawin (1994b). It demonstrates that the increase in directly recorded afferent cardiac nerve activity due to volume expansion and the gain of the cardiopulmonary baroreflex were impaired after myocardial infarction with overt congestive heart failure. From the data, it was concluded that an impairment of intracardiac receptors in the afferent portion of the cardiopulmonary baroreceptor occurred. To date, there have been no reports that the cardiac baroreflex is also impaired centrally after myocardial infarction, but we cannot definitely exclude this possibility from our experiments.

Since we saw primary pathological changes in the left ventricle after coronary artery ligature, we might assume altered stretch sensitivity of cardiac mechanoreceptors. The intrinsic stimulus for vagal afferent fibers likely consists of chemical stimuli of different mechanical factors (e.g., left ventricular end-diastolic pressure, coronary perfusion pressure, coronary flow, smooth muscle contraction of coronary vessels, and cardiac contractility) (Ditting et al., 2005) in the affected heart. Eventually, an altered production of the extracellular matrix during remodeling could play a role (Weber, 1997; Letavernier et al., 2012; Tomek and Bub, 2017). It is known that the specialized extracellular matrix (ECM) is associated with virtually every mechanosensory system studied (Emtage et al., 2004), although what this could mean in the context of pathologically altered cardiac mechanosensation is only partially understood (Lab, 1999; Stewart and Turner, 2021).

We observed a subtle but measurable increase in renal interstitial collagen I in rats after myocardial infarction. In a healthy kidney, collagen I is normally not expressed, but has been long recognized as one characteristic component of the extracellular matrix, increasing during worsening renal sclerosis in the glomeruli and interstitium (Glick et al., 1992; Alexakis et al., 2006).

We observed an increased baseline RSNA and an impaired sympathetic control of RSNA using the cardiopulmonary baroreceptors in our rat model of HFpEF. Concerning the detection of an increased baseline RSNA, we used a previously developed, more sophisticated method to evaluate recorded raw data (Rodionova et al., 2020b) that showed a clear increase in the number of sympathetic bursts per cardiac cycle and in time-based analysis but decreased burst amplitudes in rats with myocardial infarction. As described for a normotensive nephritis model (Rodionova et al., 2020b), these opposing alterations of RSNA-firing patterns again evidently canceled the other out due to the signal integration, rendering them undetectable by the conventional analysis method.

In the ventricles of rats, collagen type I mRNA decreased by 53% after sympathectomy (Dab et al., 2009); hence, sympathetic activity does influence collagen I production. Others demonstrated recently that sympathetic denervation led to amelioration of renal fibrosis and of cellular senescence in a model of unilateral ureteral obstruction and a model of unilateral ischemia-reperfusion injury. Interestingly, the cellular senescence of renal proximal tubular epithelial cell lines was mediated by a selective α2A-adrenergic receptor *in vitro* (Li et al., 2022). These findings provide clues to the possible neurometabolic and neuroimmune mechanisms that could explain the increase of renal collagen due to impaired control of renal sympathetic nerve activity.

Afferent renal nerve activity, which is involved in the control of efferent sympathetic nerve activity (Johns et al., 2011), was again decreased in myocardial infarction as described for nephritic rats, supporting our concept that a loss of the sympathoinhibitory control provided by afferent renal nerve pathways, rather than sympathoexcitatory effects of afferent renal nerve activity, occurred (Rodionova et al., 2020b). We have described a SP-dependent neurogenic mechanism of sympathoinhibition that evidently subserved tonically sympathoinhibitory afferent pathways (Rodionova et al., 2021a). Rats on a high-salt diet developed hypertension when renal afferent nerve pathways were interrupted by a dorsal rhizotomy, suggesting that afferent nerve fibers prevent the development of high blood pressure and increase sympathetic activity (Kopp et al., 2003). SP and calcitonin gene-related peptide (CGRP) released intrarenally from afferent nerve endings aggravated inflammatory responses in experimental nephritis (Rodionova et al., 2020a; Rodionova et al., 2020b), while CGRP release from these nerve endings could be documented to be increased under these circumstances (Rodionova et al., 2020b). Hence, although we have not directly investigated this in our model, subtle secretion of SP and CGRP from afferent nerve endings, as a sign of increased efferent paracrine activity of these fibers, thus acting in a proinflammatory and profibrotic way, could have influenced the induction of collagen I production.

New-onset HFpEF in patients compared with other forms of congestive heart failure with reduced ejection fraction was already associated with indices of increased inflammation and oxidative stress, impaired lipid metabolism, increased collagen synthesis, and downregulated nitric oxide signaling (Hage et al., 2020).

Our study might have limitations as we used a model of myocardial infarction with preserved ejection fraction. However, it might not reflect all characteristics that are typical for all aspects of HFpEF in humans.

## Perspective

A better mechanistic understanding leading to the impairment of intracardiac sensory nerve endings from tonic neurons of the nodose ganglion could be of clinical relevance, since a dysregulation of renal sympathetic activity is followed by first signs of renal interstitial sclerosis even with more subtle cardiac alterations without overt congestive heart failure.

## Data Availability

The original contributions presented in the study are included in the article/Supplementary Material; further inquiries can be directed to the corresponding author.
